# Ischemia-Modified Albumin—A Potential New Marker of Oxidative Stress in Dermatological Diseases

**DOI:** 10.3390/medicina58050669

**Published:** 2022-05-17

**Authors:** Mircea Tampa, Cristina Iulia Mitran, Madalina Irina Mitran, Andreea Amuzescu, Clara Matei, Simona Roxana Georgescu

**Affiliations:** 1 Department of Dermatology, “Carol Davila” University of Medicine and Pharmacy, 020021 Bucharest, Romania; tampa_mircea@yahoo.com (M.T.); matei_clara@yahoo.com (C.M.); srg.dermatology@gmail.com (S.R.G.); 2 Department of Dermatology, “Victor Babes” Clinical Hospital for Infectious Diseases, 030303 Bucharest, Romania; amuzescuandreea@gmail.com; 3 Department of Microbiology, “Carol Davila” University of Medicine and Pharmacy, 020021 Bucharest, Romania

**Keywords:** ischemia-modified albumin, oxidative stress, inflammatory skin disorders, hair disorders

## Abstract

There is growing evidence that oxidative stress is involved in the pathogenesis of numerous conditions, including dermatological diseases. Various markers are available to assess oxidative stress, but none of these can be considered the ideal marker. Recent studies have shown that ischemia-modified albumin (IMA) is not only an indicator of ischemia, but also a marker of oxidative stress. We have conducted a narrative review to evaluate the role of IMA in dermatological diseases. We have identified 24 original articles that evaluated IMA in skin disorders (psoriasis, acne vulgaris, hidradenitis suppurativa, urticaria, vitiligo and Behcet’s disease) and hair disorders (alopecia areata, androgenetic alopecia and telogen effluvium). The results of the studies analyzed reveal that IMA may be considered a new marker of oxidative stress in dermatological diseases and offer new insights into the pathogenesis of these disorders and the theoretical basis for the development of new, effective, targeted therapies. To the best of our knowledge, this is the first review that gathers up data on the role of IMA in dermatological diseases.

## 1. Introduction

Classically, oxidative stress represents the imbalance between pro-oxidants and antioxidants in favor of pro-oxidants [1]. In recent years, it has been suggested that oxidative stress is primarily an alteration of redox signaling [2]. Reactive oxygen species (ROS) are produced by both enzymatic and non-enzymatic reactions. ROS are generated endogenously, but there are also many exogenous sources including cigarette smoke, ultraviolet radiation, and metal-catalyzed reactions [3,4]. Infections, inflammatory processes, especially those that are chronic, ischemia, senescence, physical and psychological stress are the main factors involved in generating oxidative stress in the human body [5]. The accumulation of high amounts of ROS in the skin is associated with structural changes in cell components, the release of inflammatory cytokines, apoptosis, activation of transcription factors such as activator protein 1 (AP-1), mitogen-activated protein kinase (MAPK) and nuclear kappa factor B (NF-kB). Thus, the premises are created for the onset of pathological processes that underlie the appearance of various dermatological diseases [6,7]. Under conditions of hypoxia and ischemia, ROS produce changes in the structure of albumin, being generated an altered molecule known as ischemia-modified albumin (IMA) [8]. Therefore, IMA has been proposed as a marker for diseases that combine ischemia and oxidative stress. More and more studies support the involvement of oxidative stress in many skin conditions and open new perspectives on the pathogenic mechanisms involved. The aim of this review is to bring together data on the role of IMA as a marker of oxidative stress in dermatological diseases, offering new insights into the pathogenesis of these disorders, with potential implications for the therapeutical approach.

## 2. A Short Overview of Oxidative Stress

The superoxide anion, hydrogen peroxide, and hydroxyl radicals are the main ROS that are primarily generated in the mitochondria. Mitochondrial redox centers are found in the inner mitochondrial membrane and include four protein complexes, I, II, III and IV. Extramitochondrial sources are represented by the endoplasmic reticulum, peroxisomes, lysosomes, NADPH and xanthine oxidases [9,10].

ROS play beneficial roles in the human body, but also exert harmful effects, the concentration in which they are found having an essential role in this. Under physiological conditions, ROS are found in small amounts and participate in the defense against infections, play the role of secondary messengers, and contribute to maintaining homeostasis at the cellular level. When tissues are exposed to oxidative stress for a long time, ROS accumulate and produce tissue damage; the major molecular targets are lipids, proteins and nucleic acids. The alteration of the balance between oxidants and pro-oxidants has important consequences for the cell cycle, with implications for cell differentiation and apoptosis, and can influence and disturb many signaling pathways [11,12]. Antioxidants are the primary molecules that counteract the harmful effects of ROS [13]. The main enzymes involved in antioxidant defense are superoxide dismutase (SOD), catalase (CAT) and glutathione peroxidase (GPX), these enzymes have the role of preventing the formation of free radicals [14].

Oxidative stress produces lipid damage known as lipid peroxidation; polyunsaturated fatty acids that have a carbon double bond in their structure are most susceptible to the action of ROS. The main molecules resulting from lipid peroxidation are aldehydes, which in turn can interact with nucleic acids and proteins and produce structural and functional damage to the cells. The cell’s ability to repair the lesions may be exceeded, forcing the cells to undergo apoptosis [15,16]. Moreover, oxidative stress generates various changes in the structure of DNA, such as DNA base modifications, single- and double-strand breaks, and the formation of apurinic or apyrimidinic lesions [17]. Alterations in the structure of purine and pyrimidine bases, such as oxidation and methylation, lead to the most notable phenotypic changes [18]. In the presence of ROS, proteolysis is accelerated. Oxidation of thiol groups, aromatic hydroxylation and the formation of carbonyl groups are the main consequences of the protein oxidation. Methionine, cysteine, tryptophan and tyrosine are among the most vulnerable amino acids to the action of oxygen free radicals [19]. In addition, post-translational changes in proteins can influence cell homeostasis. Gamma-glutamylation is one of the post-translational modifications that can occur under oxidative stress conditions [20].

## 3. IMA as a Marker of Oxidative Stress

Free radicals are characterized by a very short half-life, at just a few seconds; therefore, quantification of their levels in vivo is difficult. Oxy radicals such as hydrogen peroxide have a long half-life, which varies from hours to weeks, and they can be measured more easily [21]. In most studies, the compounds that result from the action of ROS on various molecular targets (lipids, proteins, nucleic acids, etc.) are measured. Consequently, the main categories of markers of oxidative stress include markers of lipid peroxidation (malondialdehyde, 4-hydroxynonenal, thiobarbituric acid reactive substances, F2-isoprostanes), markers of protein oxidation (carbonyls, advanced oxidation protein products, 3-nitrotyrosine) and markers of nucleic acid oxidation (8-hydroxy-2′-deoxyguanosine, 7,8-dihydro-8-oxoguanozina) [21,22]. Therefore, various markers are available to assess oxidative stress, but none of them can be considered the ideal biomolecule. Recent research has indicated that IMA, a product of protein oxidation, could be a marker of oxidative stress.

Albumin is the major protein in human plasma, and its low levels have been associated with significant mortality and an increased risk of acute coronary heart disease. This observation is explained by the ability of albumin to bind toxic compounds in the bloodstream. The amino terminal end [N-terminal] of albumin is the binding site for various metals, including cobalt, copper and nickel [23]. Under ischemic/oxidative stress conditions, the N-terminal end of albumin changes its structure. In the presence of free radicals and free iron and copper ions, the cleavage of the first two residues and an oxidative process take place [24]. This variant of albumin has been called IMA [25].

Several mechanisms of IMA generation have been postulated in acute myocardial ischemia. After the rupture of the atheroma plaque, Cu^2+^ is released from proteins, reduced to Cu^+^, and in association with ascorbic acid and oxygen leads to the formation of superoxide radicals. Under the action of SOD, hydrogen peroxide is generated, which enters the Fenton reaction forming hydroxyl free radicals, which will alter various structures such as lipids and proteins including albumin. Normally, Cu^2+^ is scavenged by human serum albumin, but altered albumin loses this ability, and consequently, Cu^2+^ enters the reaction again, leading to ROS generation, and the process is perpetuated [26].

IMA exhibits a low affinity for cobalt, which can be measured through an indirect technique, albumin cobalt-binding (ACB) assay [24]. ACB testing indirectly measures the serum level of IMA. Elevated levels of IMA lead to an increased level of unbound cobalt ions, which will interact with a chromogen (dithiothreitol) that can be measured photometrically [23]. However, there are studies that show that the ACB test has some limitations. These include the lability of the reagents and the binding sites for fatty acids in the structure of albumin that may mask cobalt binding sites, which may decrease the accuracy of the test [27]. Da Silva et al. proposed a colorimetric method that uses nickel instead of cobalt ions, and they observed a significant correlation with the ACB test [28].

High levels of IMA have been associated with infections, neoplasms, liver cirrhosis, etc. [25]. On the other hand, low levels of IMA have been identified in disorders characterized by elevated lactate levels such as sepsis and renal failure [29].

## 4. Materials and Methods

We conducted a narrative review using the databases PubMed and Google Scholar and the keywords “ischemia modified albumin”, “skin diseases”, “hair diseases”, “dermatology”. We have included original articles written in English that evaluated IMA in patients with dermatological diseases. Reviews and original articles evaluating IMA in diseases other than dermatological diseases were excluded. Due to the great heterogenicity of the studies, a meta-analysis was not deemed appropriate.

## 5. Results

We have identified 24 original articles; all were published in the last decade (2012–2022). IMA has been evaluated in skin diseases (psoriasis, acne vulgaris, hidradenitis suppurativa, urticaria, vitiligo and Behcet’s disease) and hair diseases (alopecia areata, androgenetic alopecia and telogen effluvium).

Table 1 summarizes the results of the studies that included patients with skin disorders and Table 2 shows the results of studies that included patients with hair disorders.

## 6. Discussion

The skin is the interface between the human body and the external environment. Environmental factors play an essential role in inducing ROS production in the skin. At the skin level, ROS are mainly produced by keratinocytes, but in fact virtually all types of skin cells are able to produce ROS in response to various signals from aggressors such as pollutants, UV radiation, drugs or molecules including cytokines and growth factors [14]. UV radiation is one of the main sources for ROS generation. After a short exposure of approximately 15 min, measurable levels of H_2_O_2_ and OH^−^ have been identified in the skin [53]. ROS induce erythema by upregulating cyclooxygenase-2, a key enzyme involved in the synthesis of prostaglandin E2, which will trigger an inflammatory response [54]. In addition, ROS stimulate an increased production of matrix metalloproteinases by activating the MAPK pathway and the AP-1 factor. Consequently, stimulation of the MAPK signaling pathway leads to the activation of NF-kB, which interferes with the synthesis of numerous cytokines that promote chronic inflammation [55].

Under oxidative stress conditions, ROS interact with lipids in the stratum corneum resulting in the formation of lipoproxides which will disrupt the cell redox status, and ROS also promote the fragmentation and structural alteration of collagen fibers [56,57]. In the skin layers, there is a very well-represented antioxidant system including antioxidant enzymes (SOD, CAT, GPX) and antioxidant compounds (vitamins, glutathione, uric acid, etc.) that counteract the harmful effects of oxidative stress. When the balance between oxidants and antioxidants is disrupted, various pathological processes can occur [14].

### 6.1. IMA in Skin Diseases

Many skin conditions are associated with psychological stress [58,59]. It seems that chronic exposure to psychological stress induces an increased level of oxidative stress in the skin. Under such conditions, the renin–angiotensin system is activated, and angiotensin II induces NADPH-oxidase-dependent ROS synthesis in neutrophils by activating MAPK, extracellular-signal-regulated kinase (ERK), and phospholipase A2 signaling pathways [53]. The pathogenesis of psoriasis, a representative chronic inflammatory skin disease with a strong psychological component, is governed by a plethora of cytokines that promote excessive keratinocyte proliferation. Various signaling pathways are activated and the signals are subsequently transmitted to transcription factors (STAT or NF-kB), in a direct manner or via ROS [60]. In addition, under oxidative stress conditions, cell proliferation is stimulated by increased Ca^2+^ levels and phosphorylation. Conversely, severe oxidative damage is associated with mitochondrial alterations and the initiation of apoptosis [61].

It has been hypothesized that there is a close link between oxidative stress and melanocyte damage. Under oxidative stress conditions, melanocytes expose self-antigens that upregulate CD8 + T cells, which will release large amounts of TNF-alpha and IFN-gamma with deleterious consequences to melanocytes. Furthermore, oxidative stress induces the release of various chemokines from keratinocytes that exert a chemoattractant effect on T cells that accumulate in the skin, resulting in a dense inflammatory infiltrate frequently seen in vitiligo lesions [62,63].

The role of oxidative stress has been also identified in allergic diseases. The imbalance between pro-oxidants and antioxidants negatively affects the function of mast cells. ROS modulate important processes underlying the pathogenesis of urticaria such as mast cell degranulation, endothelial cell function and vascular permeability. Moreover, mast cell activation is influenced by ROS-dependent activation of protein kinase C [64].

The link between oxidative stress and skin microbiota is an increasingly studied topic. *Cutibacterium acnes* releases many molecules that exert a chemoattractant effect on neutrophils, which in turn generate high amounts of ROS to neutralize the microorganism. ROS accumulate and the inflammatory process that is initiated contributes to the development of acne lesions. It has been shown that oxidative stress is involved in the pathogenesis of acne through several pathways such as PPARs, TLRs, mTOR and the innate immune system [65]. IMA was evaluated in five studies that included 245 patients with psoriasis, and the results were similar, showing significantly higher levels in patients with psoriasis compared to the control group. The high levels of IMA can be explained by the increased oxidative stress identified among these patients. Regarding the correlation between the serum IMA levels and disease severity, usually assessed by the Psoriasis Area Severity Index (PASI), the results are contradictory. The study by Chandrashekar et al. showed a negative correlation between 25-hydroxy vitamin D and IMA levels in psoriasis patients and a positive correlation between IMA levels and PASI score [31]. Pektas et al. also obtained a positive correlation between serum IMA levels and psoriasis severity. Furthermore, there was a positive correlation between IMA and C-reactive protein [CRP] [33]. Conversely, Ozdemir et al. did not identify a positive correlation between serum IMA levels, PASI score and disease duration [30]. Isik et al. observed that IMA correlated with the duration of the disease, but not with the severity [32]. Kirmit et al. showed a negative correlation between IMA and albumin in psoriasis patients, but no correlation was found between IMA or the IMA/albumin ratio and disease severity [34].

We identified three studies that evaluated serum IMA levels in patients with acne vulgaris. The results were similar and showed higher levels among acne patients compared to the control group. Regarding the role of IMA as a marker of acne severity, the results are not clear. It seems that IMA levels can be influenced by the body’s compensatory mechanisms [36]. In the case of hidradenitis suppurativa, the two available studies show contradictory results. Balik et al. measured significantly higher serum levels of IMA in patients with hidradenitis suppurativa [38], instead Akdogan et al. did not identify significant differences between the patient group and the control group [37].

Only one study focused on the assessment of IMA in patients with vitiligo and showed higher levels compared to the control group. Atas et al. showed that IMA could be a reliable marker of oxidative stress in patients with vitiligo, with better sensitivity and specificity than other markers of oxidative stress (SOD, CAT) [39].

IMA levels were measured in both acute and chronic urticaria, and in both cases higher values were identified among patients compared to the control group. Otal et al. identified a positive correlation between neutrophil–lymphocyte ratio, thiol-disulfide homeostasis parameters, and serum IMA levels in patients with acute urticaria [40].

IMA has been evaluated in six studies that included patients with Behcet’s disease. In five studies, serum IMA levels were higher in patients compared to the control group; only one study found no significant differences between groups. Keskin et al. identified a positive correlation between IMA and CRP, suggesting the relationship between inflammation and oxidative stress in Behcet’s disease [46]. There are studies that have identified higher levels of IMA in patients with active Behcet’s disease compared to those with inactive disease [42,43]. However, Kor et al. did not reveal significant differences between patients with active and inactive disease [47]. Fouad et al. found no statistically significant correlation between IMA and clinical manifestations of Behcet’s disease such as oral ulcers, ocular ulcers or skin manifestations [45].

### 6.2. IMA in Hair Diseases

The scalp is chronically exposed to endogenous and exogenous factors that act as pro-oxidants leading to the disturbance of physiological processes [66,67]. The role of oxidative stress has been highlighted in alopecia areata [68,69], and it has recently been discussed that oxidative stress may also be involved in the pathogenesis of androgenetic alopecia [70]. In hair diseases, ROS accumulate in the hair follicles, and the antioxidant system is not able to counteract their harmful effects, resulting in premature aging of dermal papilla cells and even loss of cell functionality [71,72]. The contribution of oxidative stress to the pathogenesis of alopecia areata is confirmed by the encouraging results obtained after antioxidant therapies [73].

We identified two studies that measured serum IMA levels among patients with alopecia areata, and the authors found higher levels compared to the control group. Atas et al. found a positive correlation between the duration and severity of the disease in patients with alopecia areata [48]. They also identified a positive correlation between malondialdehyde, a lipid peroxidation marker, and IMA. Furthermore, SOD and IMA may be considered independent predictors for oxidative stress in patients with alopecia areata [48]. Conversely, Uysal et al. did not identify a correlation between IMA and the severity, duration or recurrence of the disease, in patients with alopecia areata. However, a positive correlation was observed between pull test positivity and adjusted IMA level [individual albumin/median albumin concentration of the population × IMA] [49]. In addition, Uysal et al. suggest that elevated levels of IMA identified in patients with alopecia areata compared to the control group may be a risk factor for heart disease, but further studies are needed [49].

IMA was evaluated in two studies including patients with androgenetic alopecia and the results were contradictory. Hussein et al. identified higher levels in patients compared to the control group [51], and Nazik et al. found no significant differences between groups [50]. Furthermore, Hussein et al. have shown that the levels of IMA correlate with the duration and severity of the disease in patients with androgenetic alopecia. Given that the levels were higher in obese patients compared to non-obese patients, the authors suggest that there is a link between androgenetic alopecia and metabolic diseases [51]. Savci et al. conducted the first study to evaluate serum IMA levels in patients with telogen effluvium and suggested the involvement of oxidative stress in the pathogenesis of this condition [52].

In this review, we have analyzed 19 studies evaluating IMA in skin diseases and 5 studies evaluating IMA in hair diseases. However, most of the studies included a small number of patients to understand the biological meaning of this marker. Furthermore, there are few studies to conclude the role of IMA in dermatological diseases. IMA is a non-specific marker; therefore, its generation may be influenced by many factors. To understand the potential biological implications of IMA in dermatological diseases, further studies including a higher number of participants are needed and the research should focus on the intimate mechanisms that underlie the increase in serum IMA levels in certain dermatological diseases.

## 7. Conclusions

In most of the studies analyzed in this review, the serum levels of IMA were significantly higher among patients compared to the control group; therefore, IMA may be considered a marker of oxidative stress in dermatological diseases. Some studies have evaluated the correlation between IMA levels and the severity or duration of the disease; however, the results are not conclusive and further studies including higher numbers of patients are needed. The data presented in this review support the involvement of oxidative stress in dermatological diseases and the potential role of antioxidants as adjuvant therapies.

## Figures and Tables

**Table 1:** The results of the studies that evaluated serum IMA levels in skin disorders.

Disease	Study Participants	IMA [Patients vs. Controls]	Conclusion	Reference
Psoriasis	26 patients 26 healthy subjects	Higher	Elevated levels of IMA could represent a mechanism of adaptation to chronic hypoxia and oxidative stress that are present in psoriasis.	Ozdemir et al. (2012) [30]
Psoriasis	43 patients 43 healthy subjects	Higher	IMA is a marker of oxidative stress and chronic inflammation in psoriasis.	Chandrashekar et al. (2015) [31]
Psoriasis	45 patients 44 healthy subjects	Higher	IMA can be considered a useful marker for the evaluation of oxidative stress in patients with psoriasis, especially those with a long duration of disease.	Isik et al. (2016) [32]
Psoriasis	44 patients 43 healthy subjects	Higher	In psoriasis, there are elevated levels of IMA in association with chronic inflammation.	Pektas et al. (2018) [33]
Psoriasis	87 patients 60 healthy subjects	Higher	High levels of IMA indicate elevated levels of oxidative stress in psoriasis.	Kirmit et al. (2020) [34]
Acne vulgaris	74 patients 60 healthy subjects	Higher	IMA is a marker of oxidative stress in patients with acne vulgaris.	Gurel et al. (2019) [35]
Acne vulgaris	90 patients 30 healthy subjects	Higher	In patients with acne vulgaris, pathological processes such as hypoxia and ischemia can be assessed by measuring the levels of IMA and methylarginine.	Akyurek et al. (2020) [8]
Acne vulgaris	30 patients 18 healthy subjects	Higher	Serum IMA level could be considered an independent predictor of acne vulgaris susceptibility and activity.	Ebrahim et al. (2020) [36]
Hidradenitis suppurativa	40 patients 40 healthy subjects	No significant difference	There was a significant difference in IMA levels between patients with metabolic syndrome and those without metabolic syndrome, but not between patients and controls.	Akdogan et al. (2018) [37]
Hidradenitis suppurativa	30 patients 30 healthy subjects	Higher	Oxidative stress is involved in the pathogenesis of hidradenitis suppurativa.	Balik et al. (2022) [38]
Vitiligo	60 patients 60 healthy subjects	Higher	IMA is an independent predictor of oxidative stress in patients with vitiligo.	Atas et al. (2017) [39]
Acute urticaria	37 patients 40 healthy subjects	Higher	IMA could be a marker of oxidative stress in acute urticaria.	Otal et al. (2021) [40]
Chronic urticaria	30 patients 20 healthy subjects	Higher	Oxidative stress may be involved in the pathogenesis of chronic urticaria.	Akdag et al. (2020) [41]
Behcet’s disease	28 patients 27 healthy subjects	Higher	Increased levels of IMA are the result of the inflammatory response induced by oxidative stress.	Ozyazgan et al. (2013) [42]
Behcet’s disease	26 patients 28 healthy subjects	No significant diference	IMA may represent a marker of disease complications in patiens with active disease.	Kilic et al. (2016) [43]
Behcet’s disease	93 patients 62 healthy subjects	Higher	IMA may be considered a biomarker in Bekcet’s disease.	Omma et al. (2018) [44]
Behcet’s disease	48 patients 38 healthy subjects	Higher	IMA is a marker of oxidative stress and disease activity in patients with Behcet’s disease.	Fouad et al. (2019) [45]
Bechcet’s disease	57 patients 45 healthy subjects	Higher	IMA is a more reliable marker to assess oxidative stress than total oxidant status, total antioxidant capacity and oxidative stress index.	Keskin et al. (2019) [46]
Behcet’s disease	39 patients 40 healthy subjects	Higher	IMA may be used as a marker of oxidative stress in patients with Behcet’s disease.	Kor et al. (2022) [47]

IMA—Ischemia-modified albumin.

**Table 2:** The results of the studies that evaluated serum IMA levels in hair disorders.

Disease	Study Participants	IMA [Patients vs. Controls]	Conclusion	Reference
Alopecia areata	60 patients 60 healthy subjects	Higher	IMA is a potential biomarker of oxidative stress in alopecia areata.	Atas et al. (2019) [48]
Alopecia areata	35 patients 35 healthy subjects	Higher	IMA may be a marker of disease activity in patients with alopecia areata.	Uysal et al. (2019) [49]
Androgenetic alopecia	50 patients 30 healthy subjects	No significant difference	In early onset androgenetic alopecia, there are no significant changes in IMA levels.	Nazik et al. (2017) [50]
Androgenetic alopecia	30 obese patients with androgenetic alopecia, 30 non-obese patients with androgenetic alopecia, 10 obese subjects with no other diseases 10 healthy subjects	Higher	IMA levels were higher in obese patients and the co-existence of androgenetic alopecia augmented the increase.	Hussein et al. (2021) [51]
Telogen effluvium	91 patients 35 healthy subjects	Higher	Oxidative stress is involved in the pathogenesis of effluvium telogen and antioxidant therapy may be useful.	Savci et al. (2020) [52]

IMA—Ischemia-modified albumin.

## Data Availability

Not applicable.
